# Complete Genome Sequences of Three *Clostridiales* R-7 Group Strains Isolated from the Bovine Rumen in New Zealand

**DOI:** 10.1128/MRA.00310-21

**Published:** 2021-07-01

**Authors:** Sam C. Mahoney-Kurpe, Nikola Palevich, Samantha J. Noel, Sandeep Kumar, Dragana Gagic, Patrick J. Biggs, Peter H. Janssen, Graeme T. Attwood, Christina D. Moon

**Affiliations:** a AgResearch Ltd., Grasslands Research Centre, Palmerston North, New Zealand; b School of Fundamental Sciences, Massey University, Palmerston North, New Zealand; c School of Veterinary Science, Massey University, Palmerston North, New Zealand; Loyola University Chicago

## Abstract

Members of the *Clostridiales* R-7 group are abundant bacterial residents of the rumen microbiome; however, they are poorly characterized. We report the complete genome sequences of three members of the R-7 group, FE2010, FE2011, and XBB3002, isolated from the ruminal contents of pasture-grazed dairy cows in New Zealand.

## ANNOUNCEMENT

The *Clostridiales* R-7 group is an abundant, but poorly characterized, group of unclassified rumen bacteria (1). Draft genome sequences of only two strains are currently available (2). The genomic characterization of additional members will accelerate efforts to understand the roles of the R-7 group in the rumen microbial ecosystem.

Rumen contents of fistulated dairy cows grazing ryegrass/clover pastures in Waikato, New Zealand (3), were obtained with AgResearch Grasslands Animal Ethics Committee approval (AE12174). An anaerobic dilution-to-extinction approach (4) was used to isolate FE2010 and FE2011 in RM02 medium supplemented with glucose, cellobiose, xylose, l-arabinose, lactate, Casamino Acids, Bacto peptone, and yeast extract (5), and XBB3002 was isolated in basal medium with yeast extract (BY) (6) at 39°C. FE2010 and FE2011 cells were Gram-negative rods, whereas XBB3002 cells were coccobacilli. Partial 16S rRNA gene sequences exhibited >96% nucleotide identity to rumen strain R-7 (7).

High-molecular-weight genomic DNA was extracted using a chemical/enzymatic lysis and phenol-chloroform extraction method (8) from 1- to 2-day-old cultures grown anaerobically in BY at 39°C. DNA was sequenced and assembled by Nextomics Biosciences (Wuhan, China). Long-read libraries were prepared using the native barcoding expansion (NBD-104) and SQK-LSK109 ligation sequencing kits and sequenced on a PromethION instrument, using Guppy v4.0.11 (Oxford Nanopore Technologies [ONT]) for base-calling and quality-filtering (Q, >7; sequence length, >1,000 bp). Short-read (2 × 150-bp) libraries were prepared using the MGISEQ-2000RS kit and sequenced using an MGISEQ-2000 instrument. Short reads were quality-filtered with fastp v0.20.0 (9); following removal of adapters, reads containing N base calls were removed. Reads had 5 bp trimmed from each end, and read pairs for which at least one read had >20% of bases with Q of <20 were removed. Quality-filtered ONT reads were assembled using Flye v2.7 (–plasmid and –nano-raw settings) (10). Assemblies were polished with Racon v1.4.13 (default settings) (11) using alignments of ONT data and with Pilon v1.23 (default settings) (12) and NextPolish v1.2.4 (default settings) (13) using alignments of the short-read data. Contigs were confirmed as circular using Circlator v1.5.1 (with the “fixstart” parameter) (14). Annotation was performed using the NCBI PGAP pipeline v5.0 (15). Sequences from each isolate assembled into circular contigs of similar size and G+C content (Table 1).

**TABLE 1:** Genome details of R-7 group strains

Parameter	Data for strain		
	FE2010	FE2011	XBB3002
BioProject accession no.	PRJNA695064	PRJNA695064	PRJNA695064
BioSample accession no.	SAMN17600269	SAMN17611198	SAMN17611949
GenBank accession no.	CP069593	CP069418	CP069419
SRA accession no. (ONT)	SRX10247579	SRX10248369	SRX10248392
SRA accession no. (MGISEQ)	SRX10247580	SRX10248370	SRX10248393
No. of raw ONT reads	815,513	966,753	443,226
No. of filtered ONT reads	781,555	926,448	419,863
*N*_50_ of filtered ONT reads (bp)	4,724	3,808	6,131
No. of raw MGISEQ reads	6,874,216	6,886,570	6,896,410
No. of filtered MGISEQ reads	6,866,512	6,878,218	6,888,148
Genome size (Mb)	3.51	3.56	3.26
No. of contigs	1	1	1
Sequencing coverage (×)	650	628	457
G+C content (%)	53.2	53.2	56.5

Taxonomic assignments were determined using GTDB-Tk v1.3.0 (16) to query the Genome Taxonomy Database (GTDB) framework (17) (release 05-RS95). The strains were classified as members of the recently proposed order “*Christensenellales*” (17) in an undescribed family (CAG-74) and genus (GCA-900199385), which the previously sequenced R-7 group strains, R-7 (species-level taxon sp900199385) and WTE2008 (sp900176495) (2), have also been classified as. FE2010 and FE2011 were assigned to the species-level taxon sp900322155, while XBB3002 was unassigned at the species level. These genomes expand the number of sequenced representatives of the R-7 group and will progress our understanding of their biology.

### Data availability.

The complete genomes and raw sequence reads are available in GenBank and the Sequence Read Archive under the accession numbers in Table 1.
